# P04.23. A survey to explore the views and practices of CAM practitioners in the UK

**DOI:** 10.1186/1472-6882-12-S1-P293

**Published:** 2012-06-12

**Authors:** A Majumdar, S Williams, N Adams

**Affiliations:** 1 London Metropolitan University, London, United Kingdom; 2 Northumbria University, Newcastle, United Kingdom

## Purpose

It is currently a transitional time for healthcare and the National Health Service in the UK, making the nature of future healthcare provision and the integration of CAM uncertain. Some studies on effectiveness of CAM for particular conditions are criticised for not representing true practice in terms of conditions treated and methods used. However, there is little extant of literature on the nature of the practice of many CAM therapies in the UK. This study is aimed to provide insight on the views and practices of CAM therapists including homeopaths, acupuncturists, naturopaths and nutritional therapists in the UK.

## Methods

Practising homeopaths, acupuncturists, naturopaths and nutritional therapists that were registered with selected key professional affiliations, from 6 geographical areas of the UK, were included in the study. A questionnaire specifically designed for the study was distributed via post or email to a randomly generated sample of registered practitioners, including n=400 homeopaths, n=800 acupuncturists and n=300 naturopaths and nutritional therapists. The data was analysed using appropriate descriptive and inferential statistics. Areas covered included details of practice, conditions encountered, methods used and opinion on medical systems. Ethical approval was sought and obtained from London Metropolitan University and Liverpool JMU ethics committees.

## Results

Conditions treated were most commonly chronic conditions, though there was some difference between those most commonly encountered between the different therapies. Similarities and differences within different groups in each profession were apparent between medical and non-medical practitioners, including consultation times, diagnostics and opinions on medical systems.

## Conclusion

Findings from the study will inform the integration debate and healthcare models of the future. They will also allow future studies on the included treatments to focus on the most relevant areas.

